# First Evidence of Cotinine in Canine Semen Reveals Tobacco Smoke Exposure

**DOI:** 10.3390/vetsci11120598

**Published:** 2024-11-26

**Authors:** Debora Groppetti, Giulia Pizzi, Elisa Giussani, Alessandro Pecile, Silvia Michela Mazzola, Valerio Bronzo, Eleonora Fusi

**Affiliations:** 1 Department of Veterinary Medicine and Animal Sciences, Università degli Studi di Milano, 26900 Lodi, Italy; debora.groppetti@unimi.it (D.G.); elisa.giussani@unimi.it (E.G.); alessandro.pecile@unimi.it (A.P.); valerio.bronzo@unimi.it (V.B.); eleonora.fusi@unimi.it (E.F.); 2 Private Practitioner, Specialist in Animal Reproduction, 20841 Carate Brianza, MB, Italy; giulia.pizzi3@gmail.com

**Keywords:** cigarette, dog, oxidative stress, sperm concentration, smoking

## Abstract

Tobacco smoke has numerous adverse effects on both human and animal health, including impaired reproductive function. Recent research has explored environmental exposure in dogs, investigating various biological matrices. However, no data are currently available on the presence of cotinine, a nicotine metabolite, in the canine ejaculate. This study aimed to evaluate the detectability of cotinine in the semen of dogs living with smoking owners. Additionally, seminal cotinine concentrations were correlated with those in serum and hair. To further examine the potential impact of smoking on canine fertility, the relationships between seminal cotinine, total sperm concentration, and antioxidant activity in plasma and semen were analyzed in exposed and non-exposed dogs. This study is the first to demonstrate the presence of cotinine in canine ejaculate and its correlation with blood and hair concentrations. While the potential toxic effect of cotinine on seminal parameters and male fertility in dogs requires further investigation, it is crucial to raise awareness among pet owners about the risks associated with domestic smoking for their animals.

## Simple Summary

In men, cigarette components have been shown to cross the blood–testis barrier, as they are detectable in the ejaculate of both active and passive smokers. However, no such data exist for dogs. To fill this gap, we investigated the presence of cotinine—a biomarker of nicotine exposure—in the ejaculate of dogs living with both smoking and non-smoking owners. Cotinine was detected in all samples, with significantly higher concentrations in dogs exposed to tobacco smoke (S) compared to non-exposed dogs (N; *p* = 0.0002). Moreover, seminal cotinine levels positively correlated with cotinine levels in blood and hair (*p* < 0.0001). We also examined the relationship between seminal cotinine concentration, total sperm concentration, and total antioxidant capacity in both blood and semen, which were similar in the two groups (S and N). The detection of cotinine in canine ejaculate confirms its utility as a biomarker of environmental tobacco smoke exposure in this species.

## 1. Introduction

Cigarette smoking has detrimental effects on reproductive health in both males and females and is also linked to various cancers, as well as respiratory, cardiovascular, and metabolic diseases [1]. In humans, smoking is associated with fertility issues and poor semen quality [2,3,4,5]. Cigarette components can cross the blood–testis barrier, leading to varying degrees of damage to germ cells [6]. Although the precise mechanisms by which cigarette smoke impairs male fertility are not fully understood, oxidative stress is one of the most widely supported hypotheses [7]. Not only does active smoking pose a risk, but exposure to secondhand smoke also presents similar reproductive hazards [8,9].

Cotinine, the primary metabolite of nicotine, is a reliable biomarker of tobacco smoke exposure. It has been detected in various human and canine biological matrices, including hair, blood, saliva, urine, and amniotic fluid [10,11,12]. While cotinine has been identified in human ejaculate, no studies have yet demonstrated its presence in dogs [13,14]. To address this gap, our study aimed to assess the presence of cotinine in the ejaculate of dogs living with (S) and without (N) smoking owners. Additionally, we correlated seminal cotinine concentrations with those in serum and hair. The serum and hair cotinine concentrations from the same subjects were previously reported by our group [15], and in this study, these were compared to seminal cotinine levels.

Furthermore, we explored the relationship between seminal cotinine concentration, total sperm concentration (TSC), and total antioxidant capacity (TAC) in both blood and ejaculate as markers of oxidative stress in exposed (S) and non-exposed (N) dogs. We hypothesized that cotinine would be detectable in all ejaculate samples, with higher levels in the S group compared to the N group and that dogs exposed to tobacco smoke would show a decrease in both TSC and TAC, as observed in human studies.

## 2. Materials and Methods

### 2.1. Animals

This study is part of a larger project investigating the effects of environmental pollutants on canine reproduction, which was approved by the Ethical Committee of the Università degli Studi di Milano (protocol OPBA_161_2019). Serum and hair cotinine concentrations used in this study were derived from aliquots of the same samples previously published [15]. In contrast, the present study focuses on their correlation with seminal cotinine concentrations.

Twenty purebred male dogs from the Veterinary Teaching Hospital of the University of Milan were enrolled in the study with the owners’ consent. Detailed information on breed, age, body weight, diet, lifestyle, and drug administration was collected for each animal. Only dogs living indoors were included in this study. Based on the smoking habits of their owners, the dogs were divided into two groups: those exposed to tobacco smoke from at least one cigarette per day over the past two months (S), and those not exposed to tobacco smoke (N). For passive-smoker dogs (S), an exposure intensity score was assigned based on the number of cigarettes smoked by the owner at home per day: “low” (one cigarette), “medium” (up to ten cigarettes), and “high” (more than ten cigarettes).

### 2.2. Specimen Collection

Blood samples (1.5 mL) were collected from the cephalic or saphenous veins into K_2_EDTA and serum separator tubes (Fisher Scientific Italia, Milano, Italy). Samples were then centrifuged (5 min at 3500× *g*) to separate plasma and serum, which were stored at −20 °C for total antioxidant capacity (TAC) and cotinine analysis, respectively. Semen collection was performed via manual stimulation [16], separating the second fraction, from which the sperm concentration was measured using a specialized canine photometer (SDM1, Minitube, Tiefenbach, Germany). Total sperm concentration (TSC) was calculated by multiplying the sperm concentration by the volume of the second fraction. Seminal plasma was obtained by centrifuging the second fraction at room temperature at 800× *g* for 10 min. The supernatant was then aliquoted and stored at −20 °C until cotinine and TAC analysis. Hair samples were clipped close to the skin in the forelimb area before blood collection and stored in paper envelopes until analysis [15].

### 2.3. Cotinine Analysis

Cotinine concentrations in serum and seminal plasma were analyzed using an ELISA assay kit (CET058Ge, Cloud Clone, Katy, TX, USA). For hair cotinine analysis, extraction was required as previously described [15]. Calibration curves were generated according to the manufacturer’s instructions (intra-assay CV < 10%; inter-assay CV < 12%).

### 2.4. Total Antioxidant Capacity (TAC) Analysis

TAC in blood plasma and seminal plasma was measured as Trolox-equivalent antioxidant capacity (TEAC) following the method described by Re et al. (1999) [17]. The TEAC method evaluates the ability of antioxidants to neutralize a stable radical cation, 2.2′-azino-bis-(3-ethylbenzothiazoline-6-sulfonic acid) (ABTS, Sigma Chemical Co, St. Louis, MO, USA). Briefly, activated ABTS solution was diluted with PBS (pH 7.4) to an absorbance of 0.70 at 734 nm and equilibrated at room temperature. Trolox standards (final concentration 0–15 mM) were prepared in PBS. Sample absorbance at 734 nm was measured after 6 min using a V-630 spectrophotometer (Jasco, Tokyo, Japan). TAC results were expressed as µmol Trolox equivalents per liter (µmol Trolox eq/L).

### 2.5. Statistical Analysis

Data analysis was performed using IBM SPSS 28.0 statistical software (IBM Italia, Segrate, Milano, Italy). Descriptive statistics for quantitative variables were reported as range, mean, and standard deviation. Since continuous variables were not normally distributed, and considering the sample size, the Mann–Whitney non-parametric test was used to compare cotinine concentrations in semen and across different matrices, as well as total sperm concentration and total antioxidant capacity in blood plasma and semen between passive smokers (S) and non-exposed (N) groups. The non-parametric Spearman correlation coefficient was employed to assess the association between seminal cotinine concentration and total antioxidant capacity. For statistical analysis, the “low” exposure intensity score (n = 1 dog) was merged with the “medium” intensity score group (n = 4 dogs). Groups (i.e., non-exposed, low–medium, and high exposure) were compared using the Kruskal–Wallis test. A cut-off value of seminal cotinine to differentiate between exposed and non-exposed dogs was determined using a receiver operating characteristic (ROC) curve with area under the curve (AUC) analysis. To verify the robustness of using cotinine in canine ejaculate as a biomarker for tobacco smoke exposure in dogs, means observed in exposed and non-exposed animals were compared using G*Power software (ver. 3.1.9.6; Heinrich-Heine-Universität Düsseldorf, Düsseldorf, Germany). A Wilcoxon–Mann–Whitney test for two independent groups was used, with an alfa error of 0.05 and a power of 0.80.

## 3. Results

All dogs included in this study were purebred, representing 13 different breeds. The dogs’ ages ranged from 1.5 to 7.5 years (mean ± SD: 4.13 ± 1.75 years), and their body weights (BW) varied from 16 to 77.7 kg (34.21 ± 12.82 kg). Based on the smoking habits of their owners, ten dogs were categorized as passive smokers (S), while the remaining ten were considered non-exposed (N). One dog was exposed to low-intensity tobacco smoke, four were exposed to medium-intensity smoke, and five were exposed to high-intensity smoke.

All dogs were fed commercial dry food; none received medications or dietary supplements. No significant differences in age (S: 3.6 ± 1.3 years, N: 4.6 ± 2.1 years, *p* = 0.4) or body weight (S: 36.1 ± 15.9 kg, N: 32.3 ± 9.2 kg, *p* = 1.0) were observed between the two groups.

**Figure 1:**
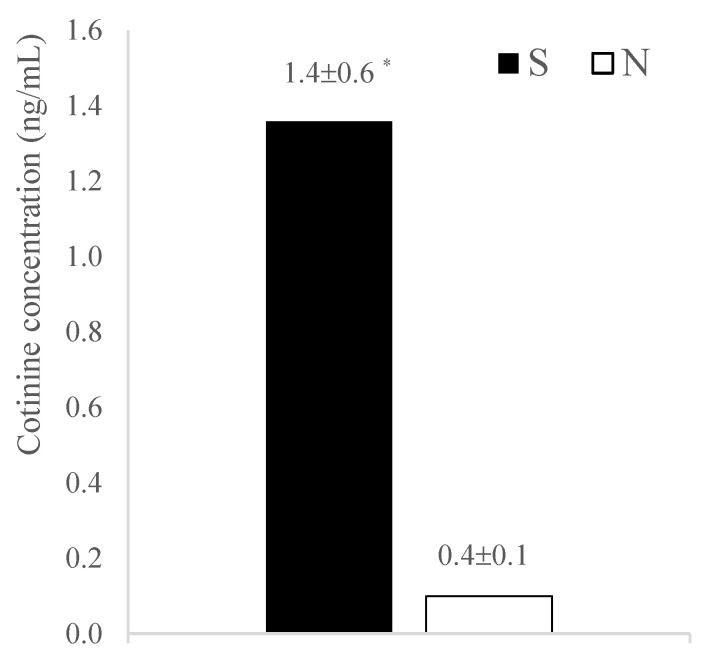
Comparison of seminal cotinine concentration between passive smokers and non-exposed dogs. S: passive-smoker dogs, N: non-exposed dogs, * indicates *p* = 0.0002.

Cotinine was detectable in the ejaculate samples of both the S and N groups, with significantly higher concentrations in dogs exposed to passive smoke compared to non-exposed dogs (*p* = 0.0002; Figure 1).

**Figure 2:**
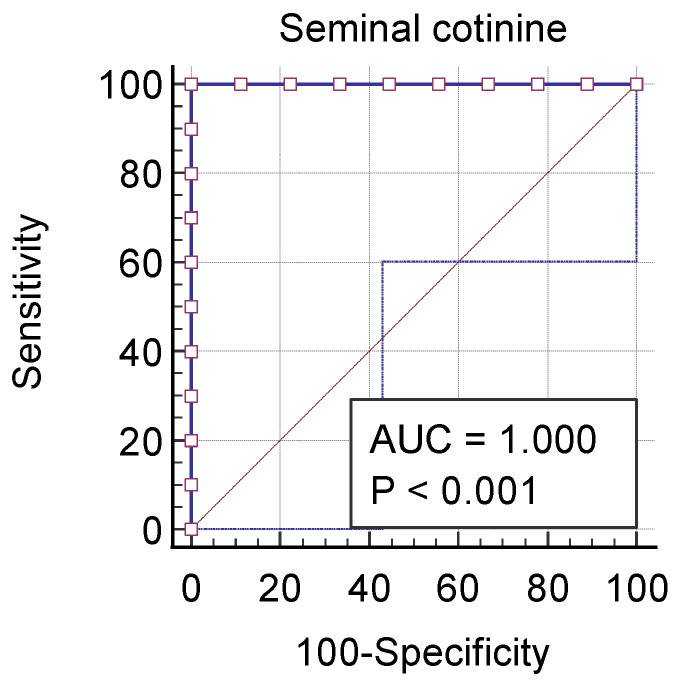
Receiver operating characteristic (ROC) curves illustrating the relationship between seminal cotinine in exposed and non-exposed dogs. ROC curves describe the tradeoff between sensitivity and specificity. The 45° diagonal of the ROC space is the random chance line. The respective area under the curve (AUC) values and level of significance are reported in the plot for each curve.

A cut-off value of 0.54 ng/mL cotinine concentration in ejaculate was able to differentiate between dogs exposed to second-hand smoke and non-exposed dogs, with both a sensitivity and specificity of 100% (Figure 2).

**Figure 3:**
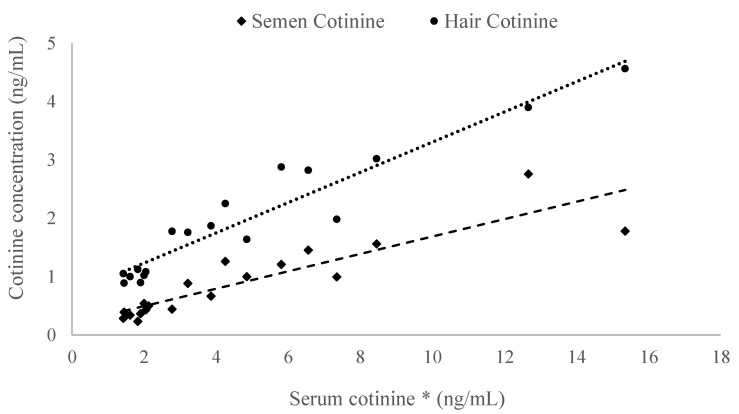
Correlation between cotinine concentrations in serum, semen, and hair. * indicates *p* < 0.0001.

Among all biological matrices analyzed in the S and N groups, the highest cotinine concentrations were found in serum, with an average of 4.7 ± 3.9 ng/mL. Serum cotinine levels were positively correlated with those in hair (1.9 ± 1.07 ng/mL, *p* < 0.0001) and semen (0.9 ± 0.6 ng/mL, *p* < 0.0001) (Figure 3).

The intensity of exposure, measured by the number of cigarettes smoked per day by the owner, did not significantly affect the concentration of cotinine in the ejaculate. The cotinine levels for dogs with low–medium exposure were 1.30 ± 0.8 ng/mL, and for those with high exposure were 1.41 ± 0.3 ng/mL (*p* = 0.694). The performed power analysis, based on observed means of cotinine in canine ejaculate in two experimental groups, identified five dogs in each group, confirming semen cotinine as a robust biomarker.

**Figure 4:**
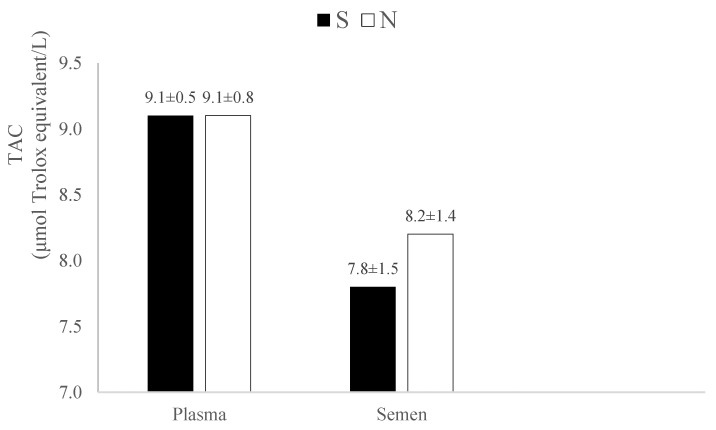
Total antioxidant capacity (TAC) in plasma and semen. S: passive-smoker dogs; N: non-exposed dogs.

Total sperm concentration (TSC) ranged from 86 × 10^6^ to 1370 × 10^6^ spermatozoa, with no significant difference between the S and N groups (*p* = 0.5). However, the average TSC was lower in dogs exposed to passive smoke (620.7 ± 432.1 × 10^6^) compared to non-exposed dogs (751.8 ± 419.1 × 10^6^), though this difference was not statistically significant. The total antioxidant capacity (TAC) in plasma was nearly identical between passive-smoke-exposed dogs (9.11 ± 0.5 μmol Trolox equivalent/L) and non-exposed dogs (9.11 ± 0.8 μmol Trolox equivalent/L). In seminal plasma, TAC was slightly lower in dogs exposed to passive smoke (7.18 ± 1.5 μmol Trolox equivalent/L) compared to non-exposed dogs (8.2 ± 1.4 μmol Trolox equivalent/L), though this difference was not statistically significant (Figure 4).

## 4. Discussion

While cotinine has previously been detected in various biological matrices of dogs living indoors with smoking owners [12,18,19,20], no data were available on its presence in canine semen until now. In humans, cotinine has been found in the ejaculate of both active and passive smokers [10]. As expected, similar to findings in men [21], cotinine was detectable in canine semen, suggesting its transfer across the blood–testis barrier from the bloodstream to the seminiferous compartment and into the seminal plasma. The seminal cotinine concentration significantly differed between dogs exposed to passive smoke and non-exposed dogs (*p* ≤ 0.0002). In particular, passive-smoker dogs had higher seminal cotinine concentrations than non-exposed dogs, with a threshold of 0.54 ng/mL, which effectively discriminated between these groups.

A direct comparison with human data is challenging due to the limited studies available on seminal cotinine concentrations in passive smokers, as most studies focus on active smokers versus non-smokers. Moreover, these studies often employ different measurement techniques, sometimes based on older data. Additionally, species-specific differences in cotinine metabolism, influenced by varying enzyme activities, may account for differences [22]. However, the average seminal cotinine values in dogs exposed to smoke (1.4 ng/mL) are comparable to those reported in passive-smoker men (0.86–2.2 ng/mL) by Pacifici et al. [9] but lower than those reported by Terzioğlu et al. [23] (8.01 ng/mL), though the latter had a highly variable range (standard deviation 21.12). Cotinine concentrations in non-smokers’ semen also vary significantly in the literature, ranging from 0.8 ng/mL (Vine 1993)—a value comparable to our findings in non-exposed dogs (0.4 ng/mL)—to 7–12 ng/mL [23,24].

In humans, cotinine can impair seminal parameters and reproductive performance, affecting erectile function, libido, sperm concentration, and the percentage of motile and morphologically normal spermatozoa [2,3,4,5]. This can occur through the disruption of spermatogenesis or altered hormone production [9,10,25,26]. In our study, total sperm concentration (TSC) was slightly lower in passive-smoker dogs than in non-exposed dogs, though the difference was not statistically significant. Despite the similar age and body weight between groups, multiple factors could influence this parameter, warranting further investigation to clarify the relationship between smoking and TSC.

Total antioxidant capacity (TAC) assays are widely used to assess oxidative status in biological samples, including canine plasma and semen [27,28,29,30]. The impact of active and passive smoking on TAC remains unclear, as studies have yielded conflicting results. Some human studies are biased by focusing on infertile patients, where other factors may influence low seminal TAC values [31]. Furthermore, various analytical methods using different units of measurement have made it difficult to review the literature critically. Generally, seminal TAC tends to be higher in non-smokers than smokers [32,33,34]. At the same time, serum TAC results are inconsistent, with some studies reporting higher values in smokers [35] and others in non-smokers [36].

In our study, TAC was measured using the Trolox equivalent antioxidant capacity (TEAC) assay, a method previously applied in humans and other animals such as horses [37], pigs [38], boars [39], and dogs [29,40]. Similar to humans, TAC values in our study were slightly higher in the ejaculate of non-exposed dogs compared to passive-smoker dogs. However, the difference was not statistically significant, suggesting that smoking may be associated with reduced antioxidant capacity. The literature presents conflicting data, with higher Trolox equivalent/L concentrations reported in the serum of non-smokers compared to smokers and passive smokers in some studies [36,41,42]. In contrast, others report higher TEAC levels in smokers [35,43]. In our study, plasma TAC values were similar between exposed (S) and non-exposed (N) dogs.

Lastly, seminal cotinine concentration was not influenced by the number of cigarettes smoked by the owner, potentially due to the limited sample size. Previous studies in dogs have shown a proportional correlation between the number of cigarettes and cotinine concentrations in serum and urine [19] and no relationship between cotinine levels in serum, hair, and amniotic fluid [12,15]. This inconsistency could be due to the unknown duration of exposure to smoke in dogs, such as the number of hours per day spent indoors, a factor considered in human studies [9].

In agreement with findings in humans [10,44], the positive correlation between cotinine concentrations in semen, blood, and hair observed in our study supports the use of these matrices interchangeably for diagnostic purposes.

## 5. Conclusions

Despite the small sample size, cotinine was confirmed as a robust biomarker of tobacco smoke exposure in canine ejaculate. Future studies are required to further investigate the impact of cotinine on seminal parameters, oxidative balance, and male fertility in dogs. Additionally, to better understand cotinine exposure in companion animals, future research should consider environmental variables such as household size, ventilation systems, and air circulation patterns, as these factors may influence cotinine levels detected in biological matrices. The presence of cotinine in semen raises concerns about its potential direct harmful effects on germ cells [45]. Assessing total antioxidant capacity (TAC), reactive oxygen species (ROS), and sperm DNA fragmentation could help clarify the effect of secondhand smoke exposure on canine fertility.

## Data Availability

The raw data supporting the conclusions of this article will be made available by the authors on request.
